# Factors Confounding the Athlete Biological Passport: A Systematic Narrative Review

**DOI:** 10.1186/s40798-021-00356-0

**Published:** 2021-09-15

**Authors:** Bastien Krumm, Raphael Faiss

**Affiliations:** 1 Institute of Sport Sciences, University of Lausanne, Lausanne, Switzerland; 2 Center of Research and Expertise in Anti-Doping Sciences - REDs, University of Lausanne, Lausanne, Switzerland

**Keywords:** Athlete biological passport, Blood passport, Haematological variables

## Abstract

**Background:**

Through longitudinal, individual and adaptive monitoring of blood biomarkers, the haematological module of the athlete biological passport (ABP) has become a valuable tool in anti-doping efforts. The composition of blood as a vector of oxygen in the human body varies in athletes with the influence of multiple intrinsic (genetic) or extrinsic (training or environmental conditions) factors. In this context, it is fundamental to establish a comprehensive understanding of the various causes that may affect blood variables and thereby alter a fair interpretation of ABP profiles.

**Methods:**

This literature review described the potential factors confounding the ABP to outline influencing factors altering haematological profiles acutely or chronically.

**Results:**

Our investigation confirmed that natural variations in ABP variables appear relatively small, likely—at least in part—because of strong human homeostasis. Furthermore, the significant effects on haematological variations of environmental conditions (e.g. exposure to heat or hypoxia) remain debatable. The current ABP paradigm seems rather robust in view of the existing literature that aims to delineate adaptive individual limits. Nevertheless, its objective sensitivity may be further improved.

**Conclusions:**

This narrative review contributes to disentangling the numerous confounding factors of the ABP to gather the available scientific evidence and help interpret individual athlete profiles.

## Key Points

- Through longitudinal, individual and adaptive monitoring of blood biomarkers, the haematological module of the athlete biological passport (ABP) has become a valuable tool in anti-doping efforts
- This literature review described the potential factors confounding the ABP to outline influencing factors altering haematological profiles acutely or chronically.
- While our results support the current ABP paradigm as rather robust to delineate adaptive individual limits, our work may contribute to disentangling the numerous confounding factors of the ABP to gather the available scientific evidence.

## Introduction

The concept of an athlete biological passport (ABP) was developed in the early 2000s and officially introduced in 2009 [1], marking a turning point in the anti-doping field. With the haematological and steroidal modules of the ABP, blood and urine variables were no longer utilised exclusively as direct screening parameters but as robust markers of either erythropoietic stimulation or steroid intake in individual, longitudinal monitoring [2]. This indirect screening approach is pertinent because indirect biological markers may reveal abnormal variations in blood and urine potentially induced by doping [3].

The ABP is implemented with an adaptive probabilistic model based on a Bayesian approach [4] to determine the probability that an athlete’s haematological variations are of prohibited origin [5]. Therefore, the effect of doping is not directly examined via the modification of the biological parameters but via the variation in the mean and standard deviation of these biomarkers [3]. When a first sample is collected, upper and lower thresholds are determined with population-based average benchmarks [5]. These individual limits are then subsequently and progressively adapted based on each athlete’s values as additional samples are taken [5].

Fourteen parameters are currently recorded and analysed as part of the ABP haematological module in the online Anti-Doping Administration and Management System (ADAMS), which was developed and supported by the World Anti-Doping Agency (WADA). In ADAMS, two primary markers are monitored with remarkably strict operating guidelines [1]: haemoglobin concentration ([Hb]) and the erythropoiesis stimulation index ‘OFF-Score’ (OFFs), calculated as OFFs = [Hb] − 60 × √Ret%, including the percentage of reticulocytes (Ret%) and [Hb] in g L^−1^. An atypical passport finding (ATPF) is generated when a primary marker value falls outside the athlete’s intra-individual range or when a longitudinal profile of primary marker values is outside expected ranges (sequence deviations), assuming a normal physiological condition’ [1]. A level of specificity of 99% (outliers correspond to the values outside the 99%-range i.e. at least 1:100 chance that this result is due to natural physiological origin) is required for the system to notify an ATPF.

The ABP uses this quantitative Bayesian model to detect an ATPF. Then, a qualitative expert review by the Athlete Passport Management Unit (APMU) handles administration of the individual passport before eventually requesting an evaluation by three independent ABP experts [4].

The latter expert review is essential because factors other than doping, such as physiological variations of biological origin or the results of an athlete’s activity [3], may explain variations in haematological biomarkers. Interestingly, the plasma volume (PV) is at the core of many haematological changes [6], and [Hb], which is, by definition, measured as a concentration, may significantly vary and become difficult to interpret if PV changes [7]. In an athletic context, red blood cells diluted in plasma will be affected differently by conditions such as acute and chronic exercise, environmental factors or certain illnesses [8], and these conditions can be defined as confounding factors leading to a potential misinterpretation of the ABP biomarker variations [9].

The variability caused by confounders can, however, be significantly reduced by understanding the nature of these factors [3]. Four main sources of variation may be outlined: pre-analytical and analytical conditions, physical exercise, environmental conditions and individual characteristics. Currently, very strict guidelines for blood collection and analyses [10, 11] address the effect of pre-analytical and analytical variations. In the current ABP operating guidelines [1], the notion of confounding factors is outlined only for the steroidal module (i.e. urine testing) of the ABP with reference to a recent review of confounders by Kuuranne et al. [12]. However, to the best of our knowledge, no systematic review has thoroughly examined the potential confounders affecting biomarkers of the haematological module. We hypothesise that the scientific evidence of confounders impacting blood profiles may challenge the current ABP approach. A review of the factors confounding hematological variables may thus help experts in their interpretation of abnormal ABP profiles.

Therefore, this systematic review investigates the existing literature regarding the various factors influencing haematological markers. Its results, presented in a narrative format, aim to enhance ABP experts’ prevailing understanding of these confounders.

## Methodological Approach

### Information Sources

**Fig. 1:**
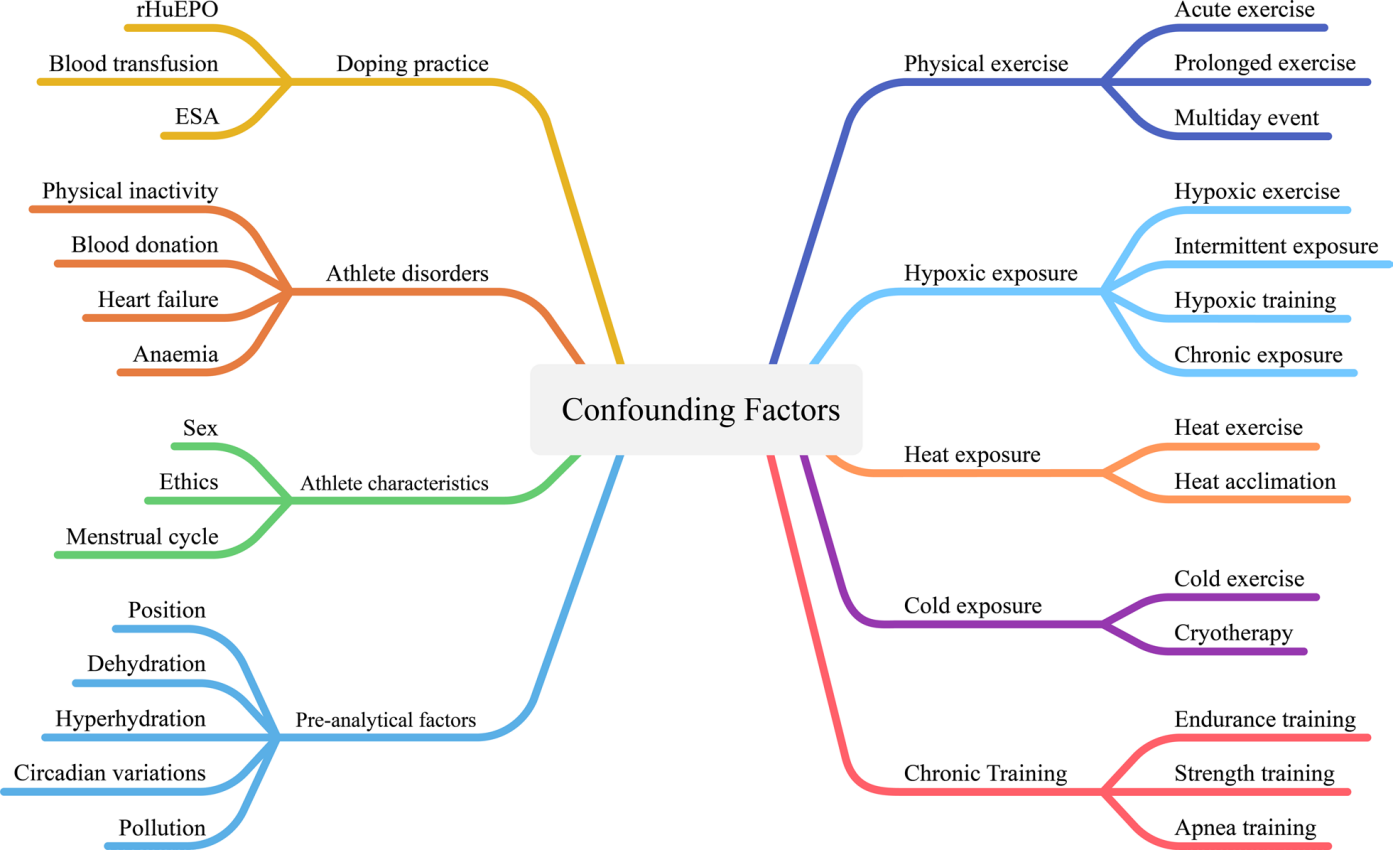
Illustration of initially identified confounding factors

Nine potential confounding factors of the haematological module were identified at the beginning of this review: doping practices, acute exercise, chronic training, exposure to a hot environment, exposure to a cold environment, exposure to a hypoxic environment, individual disorders or diseases, athlete characteristics and pre-analytical factors (Fig. 1).

A literature search was conducted in June 2020 on the PubMed and Google Scholar platforms with the above factors as research terms used in various combinations with the following terms: (haemoglobin OR haematocrit OR reticulocyte OR plasma volume) AND (haematology OR haematology OR haematological variation OR haematological parameter) AND (athlete) AND (sport).

### Study Selection

From the results, the authors selected the most relevant articles considering the broad spectrum of different confounding factors potentially involved in blood variations. The main findings are presented in the result tables. Initially, all resulting titles and abstracts (*n* = 2325) were screened with adequate articles retained for further evaluation (*n* = 535). Grey literature was included by screening the references of the most relevant articles. Only studies in English with human subjects were considered for inclusion.

### Eligibility Criteria

**Fig. 2:**
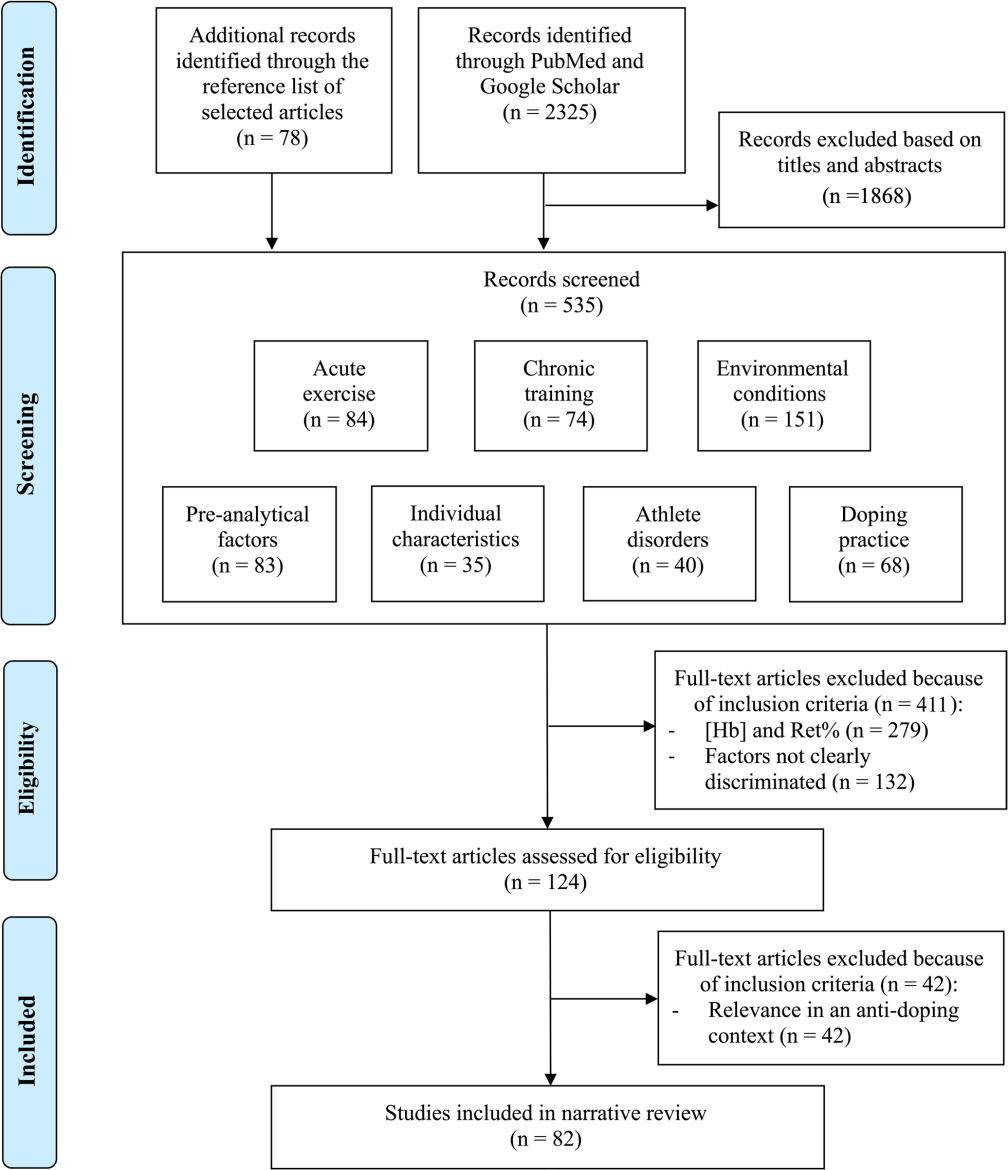
“PRISMA” flow diagram (Moher et al., 2009) Haemoglobin concentration ([Hb]); reticulocytes percentage (Ret%)

Studies where the effects of at least two confounding factors could not be clearly discriminated were excluded. Studies including both of the two primary ABP biomarkers (i.e. [Hb] and Ret%) were identified in a first selection round (*n* = 124). At this stage, however, no studies related to climatic conditions (heat and cold exposure) matched with the above-mentioned inclusion criteria, despite the fact that these conditions exhibit an impact on PV variations affecting ABP markers. Hence, several articles related to environmental conditions but reporting no direct measurements of Ret% or [Hb] were included in this review when PV changes (with a subsequent impact on [Hb]) were deemed pertinent. The process resulted in the selection of 82 pertinent studies in an anti-doping context where added value for the interpretation of ABP profiles was identified (as illustrated in the PRISMA flow diagram below in Fig. 2).

### Data Extraction

With the existence of strict WADA guidelines ruling out confounders mentioned in the doping control forms [1], this narrative review will, however, focus mainly on confounding effects not addressed by the latter WADA rules. By reviewing the multiple confounders identified (Fig. 1), some pre-analytical factors (e.g. acute exercise or exposure to various extreme environments) may exert a significant influence on the haematological biomarkers, increasing the risk of misinterpretation of blood profiles. The period of influence these factors ranges from a few minutes (e.g. body position during sampling [13]) to a few hours (e.g. intense exercise [14]), and can, therefore, alter blood variables. For this review, we have considered the relative changes observed after an intervention or in a specific condition for the following physiological variables: [Hb], Ret%, OFF-Score, haematocrit (Hct) and PV.

## Results

The literature review indicates that doping practices represent a major confounder altering the ABP haematological variables most significantly (Tables 1 and 2). Other significant changes were observed after prolonged exercise (Tables 3 and 4), exercise training (Table 5), training periodisation (Table 6), thermal acclimation (Table 7) and hypoxic training (Table 8). Athlete characteristics (Tables 9 and 10) also produced significant changes. Less evident changes occurred due to pre-analytical conditions (Table 11) and acute exercise (Table 12).

**Table 1:** Changes of haematological variables related to rhEPO doping protocol

Authors	Subjects	Interventions	[Hb]	Ret%	OFFs	Hct	PV
*rhEPO doping*							
Bejder, Aachmann-Andersen et al. [15]	Recreational athletes (*n* = 16)	Sixteen subjects received either 65 IU rhEPO·kg^−1^ every second day for 2 weeks (G1/normal-dose) or 390 IU rhEPO·kg^−1^ on 3 consecutive days (G2/high-dose) for 13 d in a randomised, placebo-controlled, double-blind crossover design	=/=	↑ + 81%	↓ − 14%	–	–
			(NS)	↑ + 135% (11 d)	↓ − 21% (11 d)		
				↓ − 26%	↑ + 18%		
				↓ − 21%	↑ + 13%		
				(25 d)	(25 d)		
Bornø et al. [16]	Recreational athletes (*n* = 24)	Twenty-four human subjects divided into three groups with eight subjects each were injected with rhEPO. The G1 and G2 groups received rhEPO for a 4-week period with 2 weeks of boosting followed by 2 weeks of maintenance and a washout period of 3 weeks. G3 received rhEPO for a 10-week period: boost (3 weeks/T1), maintenance (7 weeks/T2) and washout (1 week/T3)	=	↑	↓	–	–
			(*T1*)	(*T1*)	(*T1*)		
			↑	↓	↑		
			(*T3*)	(*T3*)	(*T3*)		
Clark et al. [17]	Recreational athletes (*n* = 16)	Eight subjects were assigned to receive six subcutaneous high-doses of 250 IU·kg^−1^ of rhEPO over 2 weeks (G1) while eight other subjects were assigned to receive six high-doses plus nine rhEPO micro-doses over a further 3 weeks (G2)	↑ + *1.5*	+ *0.7*%	↓ − *37pt*	↑ + *6*%	↓ − *6dL*
			↑ + *2.8 g·dL*^−1^	+ *1.0*%	↓ − *17pt*	↑ + *10*%	↓ − *2dL*
			(*2 weeks*)	(*2 weeks*)	↑ (*2 weeks*)	(*2 weeks*)	(*2 weeks*)
					↑ (*4 weeks*)		
Ashenden et al. [18]	Recreational athletes (*n* = 10)	Ten subjects were given twice-weekly intravenous micro-dose injections of rhEPO for up to 12 weeks. All subjects received the same total quantity of rhEPO (adjusted for body weight) during the titration phase	=	=	=	–	–
			(NS)	(NS)	(NS)		
Haile et al. [19]	Runners (*n* = 20)	Twenty well-trained Kenyan endurance runners living and training at approximately 2150 m received rhEPO injections of 50 IU·kg^−1^ body mass every 2 d for 4 weeks. This cohort (KEN) was compared with a previously published cohort (SCO) of 19 Scottish endurance-trained men based at or near sea level	↑ + 10%	↑	↓	↑ + 10%	–
			(4 weeks)	(*4 weeks*)	(*4 weeks*)	(4 weeks)	
					↑		
					(*6 weeks*)		

Numbers represent the relative changes during the most significant measurement: haemoglobin concentration ([Hb]), reticulocytes percentage (Ret%), OFF-Score (OFFS), haematocrit (Hct) and plasma volume (PV). Values in italics correspond to absolute variations

**Table 2:** Changes of haematological variables related to various doping practices

Authors	Subjects	Interventions	[Hb]	Ret%	OFFs	Hct	PV
*Blood transfusion doping*							
Damsgaard et al. [20]	Recreational athletes (*n* = 10)	Haematological parameters were measured in 10 healthy male subjects at baseline and after the withdrawal of 20% (1.3 L) of the subjects’ blood volume on d 0, + 1, + 3, + 7, + 14, + 21 and + 28 (*W*) and after reinfusion of 0.8 L of packed RBCs on d 0, + 1, + 3, + 7, + 14 and + 21 (*R*)	↓ − 14% (*W* + 1)	↑ + 45% (*W* + 1)	↓ − 38% (*W* + 1)	↓ − 15% (*W* + 1)	–
			↑ + 4% (*R* + 1)	↓ − 38% (*R* + 7)	↑ + 10% (*R* + 1)	↑ + 4% (*R* + 1)	
Lamberti et al. [21]	Recreational athletes (*n* = 24)	Blood samples were taken from the subjects at baseline and after withdrawal and reinfusion of 450 mL of refrigerated or cryopreserved blood. Measurements were taken at baseline (D-40), withdrawal (D-25), reinfusion (*D*-0) and *D* + 3, *D* + 6 and *D* + 15	↓ − 6% (− 25 d)	↑ + 57% (− 25 d)	↓ − 25% (− 25 d)	↓ − 6% (− 25 d)	–
			↑ + 6% (3 d)	↓ − 23% (6 d)	↑ + 10% (3 d)	↑ + 6% (3 d)	
Bejder et al. [22]	Cyclists (*n* = 9)	Nine highly trained male subjects donated two 450 mL blood bags each (BT) or were sham phlebotomised (PLA). Four weeks later, a 650-kcal time trial (*n* = 7) was performed 3 d before and 2 h after receiving either ~ 50% (135 mL) of the RBC or a sham transfusion	↑ + 3% (2 h)	= (NS)	–	↑ + 3% (2 h)	–
*Carbon monoxide exposure*							
Schmidt et al. [23]	Recreational athletes (*n* = 22)	Subjects inhaled a pre-determined CO bolus five times per day, starting at 8 a.m. and then every 4 h until midnight. Subjects were allowed to sleep from midnight to 8 a.m. without taking a CO bolus	↑ + 4% (2 weeks)	↑ + 16% (1 weeks)	= (NS)	↑ + 4% (2 weeks)	= (NS)
*Desmopressin use*							
Sanchis-Gomar et al. [24]	Recreational athletes (*n* = 8)	Venous blood samples were obtained from eight physically active males on two occasions. On the first occasion, the subjects ingested 1.5 L of mineral water and 4.3 μg·kg^−1^ of desmopressin (T1). On the second occasion, the subjects ingested 1.5 L of mineral water (T2)	↓ − 9% (T1)	= (NS)	↓ − 10% (T1)	↓ − 8% (T1)	–
*Xenon use*							
Dias et al. [25]	Recreational athletes (*n* = 22)	Three independent experimental protocols were completed to achieve the individual aims of this study. To determine the chronic effects, eight subjects breathed F_i_Xe 70% for 2 min on 7 consecutive days	↓ − 7% (15 d)	= (NS)	–	↓ − 8% (15 d)	↑ + 14% (15 d)

Numbers represent the relative changes during the most significant measurement: haemoglobin concentration ([Hb]), reticulocytes percentage (Ret%), OFF-Score (OFFS), haematocrit (Hct) and plasma volume (PV). Values in italics correspond to absolute variations

**Table 3:** Changes of haematological variables related to prolonged and multiday events in ambient conditions

Authors	Subjects	Interventions	[Hb]	Ret%	OFFs	Hct	PV
*Prolonged exercise in normal ambient conditions*							
Miller, Beharry et al. [26]	Triathletes (*n* = 19)	Complete blood count tests were conducted on 19 Ironman triathletes before and after an Ironman triathlon to characterise changes in haematological parameters	↓ (*2 d*)	↓ (*2 d*)	↓ (*2 d*)	↓ (*2 d*)	↑ + 16% (2 d)
Schumacher et al. [27]	Cyclists (*n* = 39)	Twenty-three male professional cyclists and 16 inactive control subjects were investigated during a 5 d road cycling stage race at sea level. Samples were obtained every morning prior to the race and between + 1 and + 3 h after the end of each stage	↓ − 6% (5 d)	= (NS)	↓ − 15% (4 d)	↓ − 5% (4 d)	↑ + 5% (5 d)
Fallon et al. [28]	Runners (*n* = 9)	Blood samples were obtained from seven male and two female participants in a 1600 km ultramarathon foot race before (T1), after + 4 d (T2) and + 11 d (T3) of running and at the end of the race (T4)	↓ − 10% (T3)	↑ + 63% (T3)	–	–	↑ + 17% (T3)
Mørkeberg et al. [29]	Cyclists (*n* = 28)	From December 2006 to November 2007, 374 blood samples and 287 urine samples were obtained from 28 elite male cyclists from the Danish cycling team, Team CSC. Seven riders were measured once before the race and twice during the Tour de France	↓ − 12% (19 d)	= (NS)	↓ (*19 d*)	↓ (*19 d*)	↑ (*19 d*)
Voss et al. [30]	Cyclists (*n* = 12)	Twelve highly trained cyclists tapered for 3 d before 6 d of simulated intense stage racing. Samples were taken in the morning and afternoon	↓ − 13% (8 d)	= (NS)	–	↓ (*8 d*)	↑ + 24% (8 d)
Corsetti et al. [31]	Cyclists (*n* = 9)	Nine professional cyclists engaged in the 2011 Giro d’Italia stage race. Haematological parameters were measured *d* − 1 (pre-race), *d* + 12 and *d* + 22 during the race	↓ − 9% (12 d)	= (NS)	–	↓ − 7% (12 d)	↑ + 2% (12 d)
Lombardi, Lanteri, Fiorella et al. [32]	Cyclists (*n* = 253)	The study population was comprised of male professional cyclists competing in the 2010 (*n* = 144) and 2012 (*n* = 109) GiroBio 10-d stage races. Blood samples were taken before the start of the race (T1), at mid-race (T2) and at the end of the race (T3). Results are the average of the two races	↓ − 9% (T2)	↓ − 6% (T2)	↓↑ (*T2*) (*T3*)	↓↑ (*T2*) (*T3*)	–
				↑ + 15% (T3)			

Numbers represent the relative changes during the most significant measurement: haemoglobin concentration ([Hb]), reticulocytes percentage (Ret%), OFF-Score (OFFS), haematocrit (Hct) and plasma volume (PV). Values in italics correspond to absolute variations

**Table 4:** Changes of haematological variables related to prolonged and multiday events in specific conditions

Authors	Subjects	Interventions	[Hb]	Ret%	OFFs	Hct	PV
*Prolonged exercise in hot conditions*							
Rama et al. [33]	Runners (*n* = 19)	The race was conducted over five stages (5 d) totalling a distance of 230 km. The daily maximum temperature ranged between 32 and 40 °C. Pre-stage blood samples were collected within the hour prior to the start of each running stage	↓ − 7% (5 d)	–	–	↓ − 8% (5 d)	↑ + 18% (5 d)
Zappe et al. [34]	Cyclists (*n* = 9)	Nine subjects were assigned to one of two experimental treatments: euhydrated (E) and hypohydrated (H). Following 20 min of a seated test in a warm environment, each subject cycled in a semi-reclining posture for 60 min at three successive intensities, representing 22% (T1), 37% (T2) and 53% (T3) of the VO_2max_	↑/↑ (*T3*)	–	–	↑/↑ (*T3*)	↓ − 11%
							↓ − 11% (T3)
Gaebelein and Senay [35]	Recreational athletes (*n* = 4)	Four males were studied during cycle ergometer exercise and stair stepping in a hot, wet environment (32 °C) after exertion. Venous blood samples were obtained 24 h before each exercise and before and at l0 min intervals during each exercise	↑ (*10 min*)	–	–	↑ (*10 min*)	↓ (*10 min*)
*Prolonged exercise in hypoxic conditions*							
Garvican-Lewis et al. [36]	Cyclists (*n* = 30)	Four teams participating in the 2013 Tour of Qinghai Lake agreed to participate in the study. Haematological variables of sea-level (SL) and altitude (ALT) cyclists were measured during a 14-d cycling race, held at 1146–4120 m, as well as during a simulated tour near sea level (SIM)	↓ − 4% (10 d)	↑ + 28%	–	↓ − 7% (6 d)	↑ + 15% (6 d)
			↓ − 8%	↑ + 24% (10 d)		↓ − 6%	↑ + 17%
			↓ − 7% (14 d)	↓ − 18%		↓ − 7% (14 d)	↑ + 16% (14 d)
				↓ − 35% (14 d)			
Schumacher et al. [37]	Cyclists (*n* = 25)	Fourteen sea-level (SL) and eleven altitude-native (ALT), highly trained athletes participated in a 14-d cycling stage race taking place at an average altitude of 2496 m above sea level (min. 1014 m, max. 4120 m). Race distances ranged between 96 and 227 km d^−1^. Blood samples were taken on *d* − 1, + 3, + 6, + 10, + 14 (SL) and − 1, + 9, + 15 (ALT)	↓ (*14 d*)	↑ (*14 d*)	↓ (*14 d*)	–	↑ (*14 d*)

Numbers represent the relative changes during the most significant measurement: haemoglobin concentration ([Hb]), reticulocytes percentage (Ret%), OFF-Score (OFFS), haematocrit (Hct) and plasma volume (PV). Values in italics correspond to absolute variations

**Table 5:** Changes of haematological variables related to various forms of chronic training

Authors	Subjects	Interventions	[Hb]	Ret%	OFFs	Hct	PV
*Endurance training*							
Spodaryk [38]	Various sports (*n* = 39)	Haematological and iron-related parameters were measured at rest in 39 male athletes from the Polish team who participated in the 1988 Seoul Olympics. The athletes were divided into two groups: endurance-trained subjects (EN: cyclists, canoeists and rowers) and strength-trained subjects (ST: wrestlers and judoka)	Lower (*EN*)	Higher (*EN*)	–	–	–
Bejder et al. [39]	Cyclists (*n* = 11)	Eleven high-level competitive cyclists were investigated for 3 weeks. After initial measurements in week 1 (T1), training load was increased ~ 250% in week 2 (T2) followed by a reversion to the baseline training load in week 3 (T3). PV and haematological variables were determined frequently during all weeks	↓ − 6% (T2)	↑ + 15% (T2)	↓ − 16% (T2)	↓ − 5% (T2)	↑ + 10% (T2)
Green et al. [40]	Recreational athletes (*n* = 8)	Eight healthy, active but untrained males volunteered for the training study. The training program involved 8 weeks of cycle exercise, with each training session lasting for 2 h and with the intensity initially set at 62% of each subject’s VO_2max_. Training was performed on a 3-l-3 cycle, with exercise conducted for 3 consecutive days followed by 1 d of inactivity and another 3 consecutive days of exercise	↓ − 4% (4 weeks)	= (NS)	–	↓ − 5% (4 weeks)	↑ + 14% (4 weeks)
Montero et al. [41]	Untrained athletes (*n* = 9)	Nine healthy, untrained volunteers underwent supervised endurance training consisting of 3–4 × 60 min cycle ergometry sessions per week for 8 weeks. Haematological markers were determined before and at weeks + 2 (T1), + 4 (T2) and + 8 (T3) of training	↓ − 8% (T1)	= (NS)	–	↓ − 7% (T1)	↑ + 16% (T1)
*Apnea training*							
Revelli et al. [42]	Divers (*n* = 6)	Six scuba divers were tested before, during and after a 14-d Guinness saturation dive (8–10 m). During the dive, athletes breathed air at 1.8–2 ATA under the control of a team of physicians. Serum parameters were measured before (T0), during (T1, T2) and after the dive (T3, T4)	= (NS)	↓ − 44% (T3)	–	↓ − 6% (T2)	–

Numbers represent the relative changes during the most significant measurement: haemoglobin concentration ([Hb]), reticulocytes percentage (Ret%), OFF-Score (OFFS), haematocrit (Hct) and plasma volume (PV). Values in italics correspond to absolute variations

**Table 6:** Changes in haematological variables related to training periodisation

Authors	Subjects	Interventions	[Hb]	Ret%	OFFs	Hct	PV
*Training content*							
Banfi and Del Fabbro [43]	Various sports (*n* = 63)	The behaviour of reticulocyte and immature reticulocyte fraction (IRF) were analysed in top-level athletes practising rugby (RUG), ski (SKI), soccer (SOC) and cycling (CYC) throughout a competitive season. Data were collected three to four times: before the start of the training period (T1), at the beginning (T2), in the middle (T3) and at the end of the competitive season (T4)	Lower (*RUG*)	Lower (*RUG*) (*CYC*) (*SOC*) Higher (*SKI*)	–	–	–
Abellan, Ventura, Pichini et al. [44]	Various sports (*n* = 246)	Haematological parameters were measured in 96 elite athletes of various sports. Elite athletes participated in different sports (swimming, synchronised swimming, taekwondo, rhythmic gymnastics, soccer, triathlon and weightlifting)	= (NS)	= (NS)	= (NS)	= (NS)	= (NS)
Malcovati et al. [45]	Football players (*n* = 923)	This study tested the effect of age, ethnicity, exercise modalities and training phases on haematologic parameters and then estimated components of variation. Differences between low- (T1), int- (T2) and high-season (T2) were analysed	↓ (*T2*)	↓ (*T2*)	–	↓ (*T2*)	–
Banfi et al. [46]	Rugby players (*n* = 19)	Blood samples were collected at four consecutive training camps during a whole competitive season—first, at the start of the training period (T1), then after the training meeting (T2), after the first part of the championships (T3) and finally, at the end of the championships (T4)	↓ − 3% (T3)	↓ − 21% (T4)	–	↓ − 5% (T3)	–
Banfi et al. [47]	Skiers (*n* = 18)	Haematological variations of elite skiers were monitored over four seasons. The study sample included 18 alpine skiers of the Italian National Alpine Ski Team (10 men and eight women). Skiers began training in July (T1) and competed from November till April (T2)	↓ (*T2*)	↑ (*T1*)	–	–	–
*Taper period*							
Mujika et al. [48]	Runners (*n* = 8)	After 15 weeks of training, athletes completed a low-volume taper, consisting of either a 75% progressive reduction in pre-taper, low-intensity continuous training or high-intensity interval training. Blood samples were obtained before (T1) and after (T2) the taper period	↓ − 3% (T2)	↑ + 44% (T2)	–	= (NS)	–
Mujika et al. [49]	Runners (*n* = 10)	After 18 weeks of training, nine male runners were assigned to a high-frequency taper (HFT) or a moderate frequency taper (MFT), consisting of training daily or resting every third day of the taper. Blood samples were obtained before (T1) and after (T2) the taper period	= (NS)	= (NS)	–	= (NS)	–

Numbers represent the relative changes during the most significant measurement: haemoglobin concentration ([Hb]), reticulocytes percentage (Ret%), OFF-Score (OFFS), haematocrit (Hct) and plasma volume (PV). Values in italics correspond to absolute variations

**Table 7:** Changes of haematological variables related to thermal acclimation protocols

Authors	Subjects	Interventions	[Hb]	Ret%	OFFs	Hct	PV
*Heat acclimation/acclimatisation*							
Costa et al. [50]	Runners (*n* = 6)	Three running exercise-heat exposures were performed at 30 °C (T1), followed by three running exercise-heat exposures at 35 °C (T2). Each running exercise-heat exposure was separated by 48 h	↓ (*T1*)	–	–	↓ (*T1*)	↑ + 7% (T1)
Loeppky [51]	Recreational athletes (*n* = 18)	PV variations were reported after 10 d of heat acclimation from studies in winter (WIN, *n* = 8) and summer (SUM, *n* = 10). In both studies, heat acclimation and dehydrating ergometer exercise were performed at 50 °C dry bulb and 26 °C wet bulb at 30% of each subject’s VO_2max_	= ↓ − 2% (10 d)	–	–	=/= (10 d)	= ↑ + 5% (10 d)
Scoon et al. [52]	Runners (*n* = 6)	Six male distance runners completed 3 weeks of post-training sauna bathing and 3 weeks of control training with a 3-week washout. During the sauna period, subjects sat in a humid sauna at 90 °C immediately post-exercise for 30 min on 12 occasions	↓ − 1.3% (3 weeks)	–	–	↓ − 2% (3 weeks)	↑ + 7% (3 weeks)
Stanley et al. [53]	Cyclists (*n* = 7)	Seven well-trained male cyclists were monitored for 35 consecutive days (17 d baseline training, 10 d training plus sauna and 8 d training). Sauna exposure consisted of 30 min (87 °C, 11% relative humidity) immediately following normal training	↓ (*4 d*)	–	–	↓ (*4 d*)	↑ + 18% (4 d)
Tebeck et al. [54]	Cyclists (*n* = 11)	Eleven cyclists completed each of two 5-d blocks of short-term heat acclimation matched for heat index (44 °C) and total exposure time (480 min). The blocks were separated by 30 d. Two protocols were applied: dry (D/43 °C and 20% humidity) and humid (H/32 °C and 80% humidity)	↓/↓ (*5 d*)	–	–	↓/↓ (*5 d*)	↑ + 5%
							↑ + 5% (5 d)
Oberholzer et al. [55]	Cyclists (*n* = 21)	Participants completed the 5½-week heat acclimation period, after which blood sampling was repeated. Blood sampling was conducted after 2 weeks into the intervention period prior to an exercise training session	= (NS)	= (NS) *Ret count	–	= (NS)	↑ + 7% (2 weeks)
*Cold acclimation/acclimatisation*							
Banfi et al. [56]	Rugby players (*n* = 10)	Ten athletes were exposed daily to whole-body cryotherapy for 5 d at the Center of Spała, Poland. During this period, moderate training (3 h daily) continued for all athletes	↓ − 2% (5 d)	= (NS)	–	= (NS)	–
Checinska-Maciejewska et al. [57]	Swimmers (*n* = 34)	Thirty-four healthy subjects (18 men and 16 women) aged 50 years swam in cold seawater during the winter season at least twice a week. The average water temperature was 10 °C in October (T1), 1 °C in January (T2) and 4 °C at the end of April (T3)	↑ + 9% (T3)	–	–	= (NS)	–

Numbers represent the relative changes during the most significant measurement: haemoglobin concentration ([Hb]), reticulocytes percentage (Ret%), OFF-Score (OFFS), haematocrit (Hct) and plasma volume (PV). Values in italics correspond to absolute variations

**Table 8:** Changes of haematological variables related to hypoxic training strategies

Authors	Subjects	Interventions	[Hb]	Ret%	OFFs	Hct	PV
*Living high-training high (LHTH) protocols*							
Bonne et al. [58]	Swimmers (*n* = 20)	One group of swimmers lived high and trained high (LHTH, *n* = 10) for three to four weeks at 2130 m or higher, while a control group (*n* = 10) completed a three-week training camp at sea level. Haematological parameters were determined weekly three times before and four times after the training camps (+ 7, + 14, + 21 and + 28 d)	= (NS)	↓ − 34% (7 d)	↑ + 22% (7 d)	↑ + 5% (7 d)	–
Garvican et al. [59]	Cyclists (*n* = 13)	Haematological parameters were measured in 13 elite cyclists during and 10 d after 3 weeks of sea level or altitude training (2760 m). The altitude group participated in a 3-week natural altitude training camp at Passo dello Stelvio, Italy, living at 2760 m and training during the majority of ride time > 1800 m for 2–6 h d^−1^	= (NS)	↑ + 25%	–	= (NS)	–
				↑ (*12 d*)			
				↓ (*31 d*)			
Wachsmuth et al. [60]	Swimmers (*n* = 27)	Twenty-five athletes trained between one and three times for 3–4 weeks at altitude training camps. Three training camp were at 2320 m (G1) in Sierra Nevada, Spain, and one training camp was at 1360 m (G2) in Pretoria, South Africa	= ↑ + 4% (4 weeks)	=/= (NS)	=/= (NS)	=/= (NS)	=/= (NS)
Ashenden et al. [61]	Endurance athletes (*n* = 23)	The haematological and physiological responses of 23 well-trained athletes (12 cyclists, 10 kayakers, 13 triathletes and 11 middle-distance runners) exposed to a simulated altitude of 2650 m for 11 ± 23 nights (ALT) were contrasted with those of healthy volunteers receiving a low dose (150 IU·kg^−1^ per week) of rEPO for 25 d (DOP)	–	↑ + 36% (17 d)	–	–	–
*Living high-training low (LHTL) protocols*							
Ashenden et al. [62]	Endurance athletes (*n* = 64)	Haematologic data were collected from three groups: 19 elite cyclists who lived and trained 2690 m above sea level for 26–31 d (LH-TH), from 39 well-trained subjects who resided at sea level but slept at a simulated altitude of 2650–3000 m for 20–23 d of consecutive or intermittent nightly exposure (LH-TL), and from six elite Kenyan runners who lived 2100 m above sea level but descended to compete at sea level competitions	↑ (*21 d*)	↑ (*21 d*)	↑ (*21 d*)	–	–
Garvican-Lewis et al. [63]	Endurance athletes (*n* = 34)	Thirty‐four endurance‐trained athletes (20 runners, 11 cyclists and three triathletes) completed 3 weeks of simulated LH-TL altitude training, accumulating an average of 14 h d^−1^ at a simulated altitude (normobaric hypoxia) of 3000 m. Athletes received either oral (ORAL), intravenous (IV) or placebo iron supplementation (PLA)	↑ + *0.9*	↓ − *0.2*%	↑ + *10 pt*	–	–
			↑ + *0.8*	↓ − *0.2*% = (*21 d*)	↑ + *8 pt*		
			↑ + *0.6 g·dL*^−1^ (*21 d*)		↑ + *9 pt* (*21 d*)		
Neya et al. [64]	Recreational athletes (*n* = 14)	Fourteen male collegiate runners were equally divided into two groups: altitude (ALT) and control (CON). Both groups spent 22 d at 1300–1800 m. The ALT group spent 10 h/night for 21 nights in simulated altitude (3000 m), while the CON group stayed at 1300 m (Takayama, Gifu, Japan)	↑ (*17 d*)	↑ (*17 d*)	–	↑ (*17 d*)	–
Voss et al. [65]	Runners (*n* = 10)	The participants spent ~ 6 h·d^−1^ at 3000–5400 m during waking hours and ~ 10 h·d^−1^ overnight at 2400–3000 m simulated altitude. Venous blood samples were collected before hypoxic exposure (B0), after + 1 (D1), + 4 (D4), + 7 (D7) and + 14 (D14) d of hypoxic exposure and again + 14 d post-exposure (P14)	↑ + *0,9 g·dL*^−1^ (*D14*)	↑ + *0.4*%	↑ (*P14*)	–	–
			↓ − *1,3 g·dL*^−1^ (*P14*)	↓ (*D7*)			
				↓ (*P14*)			
*Intermittent hypoxic exposure (IHE) or training (IHT)*							
Kasperska and Zembron-Lacny [66]	Wrestlers (*n* = 12)	Twelve wrestlers were assigned to two groups: hypoxia (sports training combined with intermittent hypoxic exposure) and control (sports training only). An approximately 1 h intermittent hypoxic exposure was performed once a day for 10 d with one day off after 6 d	= (NS)	↑ + 350% (10 d)	–	= (NS)	–
Abellan, Ventura, Remacha et al. [67]	Triathletes (*n* = 16)	Sixteen male triathletes were randomly assigned to either the intermittent hypoxia exposure group or the control normoxic group. The exposure group were exposed to simulated altitude (4000–5500 m) in a hypobaric chamber for 3 h d^−1^, 5 d a week for 4 weeks. Blood and urine samples were collected before and after the first (T0) and the final exposures (T1) and 2 weeks after the final exposure (T2)	= (NS)	= (NS)	–	= (NS)	–
Sanchez and Borrani [68]	Runners (*n* = 30)	Fifteen highly trained endurance runners completed a 6-week regimented training with three sessions per week consisting of intermittent runs (6 × work-rest ratio of 5′:5′) on a treadmill at 80–85% of maximal aerobic speed. Nine athletes (the hypoxic group) performed the exercise bouts at FI0_2_ = 10.6–11.4%, while six athletes (the normoxic group) exercised in ambient air	= (NS)	= (NS)	= (NS)	= (NS)	–

Numbers represent the relative changes during the most significant measurement: haemoglobin concentration ([Hb]), reticulocytes percentage (Ret%), OFF-Score (OFFS), haematocrit (Hct) and plasma volume (PV). Values in italics correspond to absolute variations

**Table 9:** Changes of haematological variables related to athletes’ disorders or diseases

Authors	Subjects	Interventions	[Hb]	Ret%	OFFs	Hct	PV
*Haematological disorders and medical interventions*							
Parisotto et al. [69]	Elite athletes (*n* = 1152)	Blood samples were obtained from elite athletes from 12 countries with known haematologic abnormalities (G1). The algorithm scores for these athletes were compared with those of their healthy counterparts (G2) in terms of anaemia, iron deficiency and haemoglobinopathies	Lower (*G1*)	Higher (*G1*)	Lower (*G1*)	Lower (*G1*)	-
Stangerup et al. [70]	Recreational athletes (*n* = 18)	Blood variables were measured at baseline and + 3, + 7, + 14, + 21 and + 28 d after blood donation in 18 iron-sufficient women	↓ − 8% (3 d)	↑ + 27% (7 d) *Ret count	-	↓ − 8% (3 d)	–
Meurrens [71]	Recreational athletes (*n* = 24)	Twenty-four moderately trained subjects were randomly divided into a donation (*n* = 16) and a placebo (*n* = 8) group. The three donations were spaced 3 months apart, and the recovery of endurance capacity and haematological parameters was monitored up to 1 month after the donation	↓ − 12% (2 d)	–	–	↓ − 11% (2 d)	–
Ryan et al. [72]	Recreational athletes (*n* = 7)	Haematological values were measured for seven healthy, recreationally active men before (T1), 5 h after (T2) and 5 d (T3) after 4 d of head-down tilt bed rest	↑ + 11% (T2)	= (NS) *Ret count	–	↑ + 11% (T2)	↓ − 14% (T2)

Numbers represent the relative changes during the most significant measurement: haemoglobin concentration ([Hb]), reticulocytes percentage (Ret%), OFF-Score (OFFS), haematocrit (Hct) and plasma volume (PV). Values in italics correspond to absolute variations

**Table 10:** Changes in haematological variables related to athletes’ characteristics

Authors	Subjects	Interventions	[Hb]	Ret%	OFFs	Hct	PV
*Genetic characteristics*							
Robinson et al. [73]	Track and field athletes (*n* = 3683)	Reference ranges in top‐level track and field athletes were determined for biomarkers of altered erythropoiesis considering various factors such as gender (M/F), age, endurance (E) or non‐endurance (NE) disciplines, detailed sport disciplines, the origin of the athletes (African [AFR], South America [SAF], European [EUR], Oceania [OCE]) and the time of blood sampling	Higher (*M*) (*AFR*) (*SAF*) Lower (*OCE*)	Lower (*M*)	= (NS)	–	–
Mullen et al. [74]	Recreational athletes (*n* = 17)	Seventeen women with regular menses were included. Blood samples were collected once a week for two consecutive cycles and analysed for haematological parameters. Menstrual phases were hormonally determined: follicle (T1), ovulatory (T2) and luteal phases (T3)	= (NS)	Lower − 19% (T1)	= (NS)	= (NS)	–
Lobigs [75]	Recreational athletes (*n* = 2222)	Reference intervals of healthy athletes were established for 13 haematological parameters. Participants were subsequently characterised into five major ethnic groups: Arabic (G1), Asian (G2), Black (G3), White (G4) and Mixed (G5)	Lower (*G1*)	Lower (*G3*)	–	Lower (*G1*)	–
Steiner and Wehrlin [76]	Endurance athletes (*n* = 92)	Blood parameters were measured in three endurance athlete groups: AG16 (*n* = 14), AG21 (*n* = 14) and AG28 (*n* = 16), as well as in three age-matched control groups (< 2 h endurance training per week): CG16 (*n* = 16), CG21 (*n* = 15) and CG28 (*n* = 16)	Lower − 6% (AG16)	= (NS)	= (NS)	Lower − 6% (AG16)	= (NS)
Steiner et al. [77]	Endurance athletes (*n* = 22)	Several venous blood parameters were assessed in ten male adolescent endurance athletes (five XC-skiers and five triathletes) at seven time points in 6-month intervals, resulting in a monitoring phase of 3 years	= (NS)	= (NS)	–	= (NS)	↑ (*3 y.*)

Numbers represent the relative changes during the most significant measurement: haemoglobin concentration ([Hb]), reticulocytes percentage (Ret%), OFF-Score (OFFS), haematocrit (Hct) and plasma volume (PV). Values in italics correspond to absolute variations

**Table 11:** Changes of haematological variables related to pre-analytical variations

Authors	Subjects	Interventions	[Hb]	Ret%	OFFs	Hct	PV
*Pre-analytical factors*							
Robinson et al. [73]	Track and field athletes (*n* = 3683)	Reference ranges in top‐level track and field athletes were determined for biomarkers of altered erythropoiesis considering various factors such as gender, age, endurance or non‐endurance disciplines, detailed sport disciplines, the origin of the athletes (African vs non-African) and the time of blood sampling: morning (T1), afternoon (T2) and evening (T3)	↓ (*T2*)	= (NS)	↓ (*T2*) (*T3*)	–	–
			↓ (*T3*)				
Sennels et al. [78]	Recreational athletes (*n* = 24)	Venous blood samples were obtained under standardised circumstances from 24 healthy young men every third hour through 24 h for a total of nine samples	↑ + 3% (12:00)	↑ + 5% (12:00) *Ret count	–	↑ + 3% (12:00)	–
Kuipers et al. [79]	Endurance + Recreational athletes (*n* = 7)	In seven subjects in separate experiments, 500 mL of saline were infused around 8 a.m., and blood variables were measured before and every hour thereafter until 7 h after infusion	↓ − 5% (1 h)	= (NS)	–	↓ − 4% (1 h)	–
Bejder, Hoffmann et al. [80]	Recreational athletes (*n* = 20)	Twenty subjects received rhEPO for 3 weeks. After 10 d of rhEPO washout, 10 subjects ingested a 1000 mL bolus of water. Blood variables were measured + 20, + 40, + 60 and + 80 min after ingestion	↓ − 3% (60 min)	= (NS)	↑ − 4% (40 min)	–	↑ + 4% (60 min)
Astolfi, Schumacher et al. [13]	Cyclists + Divers + Recreational athletes (*n* = 38)	Ten successive venous blood samples from 38 subjects were collected: after 10 min of normalised activity (B1), after 10 min seated (B2, typical reference sample in an anti-doping context), after a 50 m walk (B3), after an additional 5 and 10 min in a seated position (B4 and B5) and finally after 5–30 min in a supine position (B6-B10)	↑ + 3% (B1)	= (NS)	–	↑ + 4% (B1)	↓ (B1)
Schumacher et al. [81]	Endurance athletes (*n* = 15)	Fifteen endurance athletes submitted ABP blood samples in the morning before (T1) and after arrival (T2) on an 8 h flight. Two additional samples were obtained in the morning and the evening 3 d after travel. Twelve nontraveling subjects served as controls	↓ − 0.5 g·dl^−1^ (T2)	= (NS)	–	↓ (T2)	–
Alsaadi et al. [82]	Recreational athletes (*n* = 11)	Nine healthy, physically active subjects were tested twice daily—in the morning (T1) and afternoon (T2)—for 2 d before and 3 d into the celebration of Ramadan. Sample collection and all analyses were performed according to WADA technical documents	↓ − *0.4*	= (NS)	–	–	–
			↓ − *0.3 g·dl*^−1^ (*T1*/*T2*)				

Numbers represent the relative changes during the most significant measurement: haemoglobin concentration ([Hb]), reticulocytes percentage (Ret%), OFF-Score (OFFS), haematocrit (Hct) and plasma volume (PV). Values in italics correspond to absolute variations

**Table 12:** Changes of haematological variables related to acute exercises in various environmental conditions

Authors	Subjects	Interventions	[Hb]	Ret%	OFFs	Hct	PV
*Acute exercise in normal ambient conditions*							
Morici et al. [83]	Rowers (*n* = 20)	After warmup, athletes underwent 10 min of full rest during which the rowing equipment was mounted and checked. Data were collected at least 24 h after a training session (rest) and shortly after all-out rowing over 1000 m	= (NS)	↑ + 25% (10 min)	–	↑ + 7% (10 min)	↓ − 7% (10 min)
Lobigs, Sottas et al. [7]	Endurance athletes (*n* = 33)	Subjects performed an exercise cycle ergometer challenge designed to promote an acute, maximal shift in PV: 30 min maximal step‐test on a cycle ergometer (5 min steps of 25–50 W increments)	↑ (*30 min*)	–	↑ (*30 min*)	–	↓ − 17% (30 min)
Kuipers et al. [84]	Speed skaters (*n* = 277)	Haematological parameters were analysed in blood samples taken pre-competition (T1) and post-competition (T2) in elite male and female speed skaters participating in long-track ISU speed skating events	↓ − 5% (T2)	= (NS)	–	–	–
Robinson et al. [85]	Recreational athletes (*n* = 25)	Blood samples were collected before and after a controlled cycle ergometer exercise. Exercise was as follows: 15 min of warmup, then 30 min at a constant power of 70% of the maximal aerobic power and finally 15 min at maximal effort (T1). The subjects were allowed to drink water as needed	↑ + 6% (T2)	= (NS)	–	↑ + 8% (T2)	↓ − 11% (T2)
Miller, Teramoto et al. [86]	Endurance athletes (*n* = 12)	Twelve subjects underwent multiple controlled exercise trials designed to induce varying levels of PV shifts: 75%, 65% and 55% power output of their determined VO_2peak_	↑ (*10 min*)	↑ (*10 min*)	↑ (*10 min*)	↑ (*10 min*)	↓ − 7% (10 min)
*Acute exercise in hot conditions*							
Diaz et al. [87]	Recreational athletes (*n* = 5)	Each subject participated in six separate tests consisting of 45 min of rest followed by 45 min of submaximal work on a cycle ergometer at 50 °C. They worked at approximately 30% (E1) and 45% (E2). VO_2max_ was measured in each of the following postures: upright (UR), low sit (LS) and supine (SU)	↑ + 16% ↑ + 11% ↑ + 10% (E2)	–	–	↑ + 12% ↑ + 7% ↑ + 7% (E2)	↓ − 20% ↓ − 16% ↓ − 13% (E2)
Kenefick et al. [88]	Recreational athletes (*n* = 32)	Thirty-two men divided into two cohorts—euhydration (E) and hypohydration (H)—completed trials in: ambient temperature (T1), 10 °C (T2), 20 °C (T3), 30 °C (T4) and 40 °C (T5). 30 min of cycle ergometry (at 50% VO_2max_) was performed	↑/↑ (*T5*)	–	–	↑/↑ (*T5*)	↓ − 11%
							↓ − 15% (T5)
Myhre and Robinson [89]	Recreational athletes (*n* = 12)	Twelve unacclimatized men rested for 4 h in a hot environment (50 °C) with or without fluid replacement	↑ + 2% (4 h)	–	–	↑ + 6% (4 h)	↓ − 8% (4 h)
Jimenez et al. [90]	Recreational athletes (*n* = 8)	In the thermal dehydration experiment, the subjects were dehydrated for approximately 2 h by passive controlled hyperthermia in a semi-recumbent posture. For each trial, the protocol comprised three phases: a 90-min period in a thermoneutral environment (T1), a period in which subjects were exposed to a variation in body hydration (T2) and a second 90-min period in a thermoneutral environment (T3)	↑ (*T2*)	–	–	↑ (*T2*)	↓ − 12% (T2)
*Acute exercise in cold conditions*							
Vogelaere et al. [91]	Recreational athletes (*n* = 25)	Subjects began with a 30-min rest period (T1). Next, they performed either a cycle ergometer test during which they performed a 120-min sub-maximal exercise corresponding to 40% of the maximal power (SUB) or a progressively increasing workload till exhaustion characterised by an initial 60 W work (T2). Finally, subjects experienced 30 min of passive recuperation (T3)	↑ + 5% ↑ + 9% (T2)	–	–	↑ + 8% ↑ + 8% (T2)	↓ − 7% ↓ − 6% (T2)
Vogelaere et al. [92]	Recreational athletes (*n* = 6)	In a study of six young males, the experimental group, resting in a dorsal reclining position, was exposed successively to a thermoneutral environment (30 min, T1), a cold environment (1 °C; 30 min, T2), and a 60-min recovery period in thermoneutral conditions while the control group, also resting in a dorsal reclining position, was exposed to thermoneutrality (control, T3) for the entire 120 min	↑ + 4% (T2)	–	–	–	↓ − 15% (T2)
*Acute exercise in hypoxic conditions*							
Siebenmann et al. [93]	Recreational athletes (*n* = 9)	Nine healthy, normally trained, sea-level residents (eight men, one woman) sojourned for 28 d at 3454 m. They remained at that altitude for 4 weeks. Individual physical activity was maintained by hiking/mountaineering, ergometer cycling and resistance training	↑ (*4 weeks*)	↑ + 117% (9 d) *Ret count	↓ (*4 weeks*)	↑ (4 weeks)	↓ − 11% (4 d)

Numbers represent the relative changes during the most significant measurement: haemoglobin concentration ([Hb]), reticulocytes percentage (Ret%), OFF-Score (OFFS), haematocrit (Hct) and plasma volume (PV). Values in italics correspond to absolute variations

First, differences observed in [Hb] related to doping practices ranged from + 10% following rhEPO doping [19] to − 14% after blood withdrawal [20]. Otherwise, the range of increase or decrease in [Hb] was − 5% due to certain pre-analytical conditions [79], + 16% after acute exercise [87], − 13% after prolonged exercise [30], − 8% after exercise training [41], − 2% after heat acclimation [51], + 9% after cold acclimation [57], + 4% after hypoxic training [60] and − 12% after blood donation [71].

Subsequently, a large amplitude was observed in the relative changes in Ret% with increases up to + 135% after rhEPO doping [15] and decreases of − 38% observed after blood transfusions [20]. The range of increase or decrease in Ret% notwithstanding doping was + 5% due to pre-analytical conditions [78], + 25% after acute exercise [83], + 63% after prolonged exercise [28], − 44% after apnea training [42], + 350% after hypoxic training [66] and + 27% after blood donation [70].

Differences in the OFF-Score as a result of doping practices ranged from + 18% after rhEPO doping [15] to − 38% after blood transfusion [20]. The margin of increase or decrease in the OFF-Score related to other factors was − 4% for pre-analytical conditions [80], − 15% after prolonged exercise [27], − 16% after exercise training [39] and + 22% after hypoxic training [58].

Hct differences ranged from + 10% after rhEPO intake [19] to − 15% after blood transfusion [20]. The range of Hct variations related to other confounding factors was + 4% [13] and − 4% [79] for pre-analytical conditions, + 12% after acute exercise [87], − 8% after prolonged exercise [33], − 7% after exercise training [41], − 2% after heat acclimation [52], + 5% after hypoxic training [58] and + 11% [72] and − 11% [71] after blood donation.

Finally, PV increased by 14% after chronic xenon inhalation [25]. The range of increase or decrease in PV linked to other parameters was + 4% for pre-analytical conditions [80], − 20% after acute exercise [87], + 24% after prolonged exercise [30], + 16% after exercise training [41], + 18% after heat acclimation [53] and − 14% after blood donation [72].

## Discussion

Our study confirmed that the most obvious factor confounding the ABP biomarkers is blood doping. Doping protocols for erythropoiesis-stimulating substances (such as rhEPO injections) were generally structured with a treatment phase causing an increase in [Hb] and Ret% (ON-phase), followed by a reversal trend when treatment was stopped (OFF-phase) [15–17], although significant inter-individual variability was observed [16]. Following blood withdrawal there should be a decrease in [Hb] and increase in Ret%, with the opposite effect occurring after re-infusion [20, 21]. In addition, chronic exposure to low doses of carbon monoxide was recently shown to positively influence erythropoiesis and alter markers sensitive to PV variations [23, 94]. Conversely, repeated intake of desmopressin or chronic xenon inhalation induced haemodilution and decreased concentration-based biomarkers sensitive to PV shift [24, 25, 95]. However, micro-dosing doping schemes complicate the analysis of blood profiles due to limited haematological variations [22]. Indeed, the efficiency of the ABP appears significantly lower with low-volume autologous transfusion protocols due to several factors that may influence its sensitivity (e.g. timing of the sample collection) [22]. The current findings outline, for instance, the need for a strategy able to detect minor blood manipulations [96].

Numerous studies have highlighted specific blood variations as the result of pre-analytical factors including circadian modifications [73, 78] and prevention strategies such as sodium intake [79], overhydration [80] or posture adjustments [13]. In the same way, a temporary [Hb] increase caused by PV reduction was usually observed following acute exercise [7, 83, 85, 86] without affecting Ret% in most cases [84]. Similarly, acute exposure to extreme environmental conditions, such as heat [87–90], cold [91, 92] or hypoxia [93], was reported to cause transitory PV shifts. Nevertheless, the strict WADA guidelines incorporate various pre-analytical precautions (e.g. reporting any exposure to hypoxia or extreme environment and other pathological conditions) to account for possible pre-analytical variations. However, other factors are not considered in the current model and may alter a proper interpretation of ABP profiles.

Prolonged exertion may affect an athlete’s blood values with temporary but delayed effects persisting for several days [26], and these effects may occur and persist independent of environmental conditions. A progressive decrease of [Hb] was observed during multiday events (e.g. cycling stage races) [27, 29, 30, 36, 37], although other studies observed a stabilisation over time [31, 33] or even an increase towards the end of the competition [32]. The Ret% was mostly unaffected by participation in multiday events [27], although some variations (unrelated to an erythropoietic stimulation) were reported [26, 28].

In contrast to transient variations caused by acute exercise, PV was shown to expand over a few weeks of endurance training, thereby reducing [Hb] [39–41]. Interestingly, multiple studies also reported a noticeable variation in Ret% after a few weeks of training [39–41]. Furthermore, acute and chronic training loads, the competition calendar and training periodisation were shown to significantly alter blood variables included in the ABP [43, 48, 49, 97], with [Hb] decreases most likely during periods with the highest training loads [46]. In a recent 12-month longitudinal study of elite cyclists addressing the influence of training on ABP variables, no ATPF was observed, underlining the relative robustness of the ABP adaptive model in incorporating varying training loads [97].

Various environmental conditions (e.g. hypoxic or hot environments) are currently gaining popularity as additional training stimuli [98] with a putative effect on fluid balance [99]. PV increase represents one of the main physiological adaptations that occur during heat acclimation strategies [8, 100], and this increase is, in turn, often linked to [Hb] reduction [50–54]. Although PV varies after heat acclimatisation, such variation may also occur without influencing ABP values [55], depending on the type of acclimatisation strategy and subjects involved. Another form of exposure to extreme environments is dry sauna bathing [101], which resulted in some heat acclimation with a significant PV expansion (up to ~ 15%) [52, 53]. A recent systematic review minimises the confounding risk of heat acclimation because no significant change in PV variations was reported after various acclimation protocols [102, 103]. Conversely, several sessions of whole-body cryotherapy were proposed to decrease [Hb] [56].

Hypoxia is another environmental condition now widely used by athletes [104] where [Hb] may be either not [58, 59] or only slightly affected [60] depending on the timing of blood sampling after *living high-training high* (LHTH) periods. Moreover, an increase in Ret% was reported during hypoxic exposure [59, 61], while Ret% decreased upon return to a lower altitude [58]. Similar blood variations were reported following *living high-training low* (LHTL) protocols with, however, a more pronounced increase in [Hb] [62–65]. Variations due to hypoxic training observed immediately after altitude exposure were reported to persist three weeks after returning to sea level [58, 62]. In addition, intermittent hypoxic exposure (IHE) or training (IHT) may positively influence Ret% [66]. Nevertheless, the rationale for the use of IHE for erythropoietic purposes in athletes is limited [105], and the response is inconsistent when hypoxic exposure is not prolonged [67, 68]. Prolonged exposure to simulated or real altitude was reported to induce similar and prominent changes in total haemoglobin mass (Hb_mass_) [106, 107]. Nevertheless, haematological variations following hypoxic training are contradictory [59, 104] and not systematically reported [63, 108], possibly due to initial fitness, initial fatigue or iron status [36, 109]. It seems unlikely, then, that training in a hypoxic environment could lead to a misinterpretation of an athlete’s blood profiles [65] if duly reported in the doping control forms as requested by WADA.

Finally, specific individual characteristics were also shown to impact haematological components in athletes. Some haematological disorders were reported to alter [Hb], with these athletes exhibiting lower values than those of healthy athletes [69]. With some athletes suffering from haemochromatosis (a pathological condition requiring the withdrawal of large amounts of blood), [Hb] was decreased immediately after withdrawal [110] while a measurable increase in reticulocytes was sometimes delayed for a few days [70]. However, most haematological disorders observed in athletes were not associated with exceeding the limits of the model used to detect rhEPO [69]. Thus, the potential of these disorders to cause misinterpretation of the ABP profiles is limited. Finally, in contrast to Ret%, which is frequently higher among women [73, 111], [Hb] is known to be higher in men than in women. Ret% was also shown to vary during the menstrual cycles of active women with lower values reported in the follicular phase [74]. Still, most of these variations remained within the individual ABP limits. Furthermore, one should be aware that individual characteristics (e.g., sex or ethnicity) used to define initial individual limits (i.e. possible inter-individual variance) then become irrelevant since the adaptive model focuses on intra-individual variation for the correct interpretation of an athlete’s hematological profile.

Finally, while anti-doping blood samples are collected and analyzed following very strict guidelines [1], pre-analytical and analytical variations [112] should not be excluded from the studies included in this review since results for hematological variables originate from different analysers and/or varying pre-analytical procedures.

## Conclusion

As this review shows, the effects of ABP confounders vary widely in amplitude and duration depending on the type of effector (i.e. doping, environmental condition, training or pre-analytical condition). Nevertheless, the absolute effects of the factors described in this review appear to be relatively limited when taken together. Furthermore, true and systematic effects of environmental conditions (i.e. heat acclimation or hypoxic training) on haematological biomarkers remain debatable due to significant differences in individual responses [103, 104]. Studies investigating specific ABP confounders generally report variations within individual limits of the adaptive model [13, 63, 65, 97]. The scientific level of ABP experts reviewing passports and their efforts to do so with the latter factors in mind can further limit misinterpretations of ABP profiles caused by the confounding factors identified in this review. Nevertheless, many authors have noted an important inter- and intra-variability concerning haematological biomarkers [111, 112], highlighting the need to improve the sensitivity of the ABP while interpreting confounders carefully. The present review contributes to a detailed understanding of confounding factors that could affect ABP biomarkers. By reporting limited variation in haematological variables through the selected studies of this review, our findings support the blood module of the ABP as an efficient instrument to deter and indirectly detect doping. Nevertheless, further studies on the confounders that affect blood variables may contribute to improving the module and thus fighting doping more effectively.

## Data Availability

Not applicable.

## Abbreviations

ABP: Athlete biological passport
ADAMS: Anti-doping administration and management system
APMU: Athlete passport management unit
ATPF: Atypical passport finding
[Hb]: Haemoglobin concentration
Hb_mass_: Haemoglobin mass
Hct: Haematocrit
IHE: Intermittent hypoxic exposure
IHT: Intermittent hypoxic training
LHTH: Living high-training high
LHTL: Living high-training low
OFFs: OFF-score
PV: Plasma volume
Ret%: Percentage of reticulocytes
WADA: World anti-doping agency
